# Hippocampal Pathophysiology: Commonality Shared by Temporal Lobe Epilepsy and Psychiatric Disorders

**DOI:** 10.1155/2018/4852359

**Published:** 2018-01-22

**Authors:** Soichiro Nakahara, Megumi Adachi, Hiroyuki Ito, Mitsuyuki Matsumoto, Katsunori Tajinda, Theo G. M. van Erp

**Affiliations:** ^1^Department of Psychiatry and Human Behavior, University of California Irvine, Irvine, CA 92617, USA; ^2^Candidate Discovery Science Labs, Drug Discovery Research, Astellas Pharma Inc., 21 Miyukigaoka, Tsukuba, Ibaraki 305-8585, Japan; ^3^Neuroscience, La Jolla Laboratory Astellas Research Institute of America, LLC. 3565 General Atomics Court, Suite 200, San Diego, CA 92121, USA

## Abstract

Accumulating evidence points to the association of epilepsy, particularly, temporal lobe epilepsy (TLE), with psychiatric disorders, such as schizophrenia. Among these illnesses, the hippocampus is considered the regional focal point of the brain, playing an important role in cognition, psychosis, and seizure activity and potentially suggesting common etiologies and pathophysiology of TLE and schizophrenia. In the present review, we overview abnormal network connectivity between the dentate gyrus (DG) and the Cornus Ammonis area 3 (CA3) subregions of the hippocampus relative to the induction of epilepsy and schizophrenia. In light of our recent finding on the misguidance of hippocampal mossy fiber projection in the rodent model of schizophrenia, we discuss whether ectopic mossy fiber projection is a commonality in order to evoke TLE as well as symptoms related to schizophrenia.

## 1. Introduction

Numerous clinical studies report that up to 30% of individuals with epilepsy often present distinct psychiatric symptoms, such as intellectual aurae, dreamy states, complex visual illusion, and auditory hallucinations [1–10]. Epileptic individuals present psychotic symptoms with significantly higher incidence than those with other chronic medical conditions or healthy individuals. For example, a recent report demonstrated that individuals with epilepsy had a 5.5- and 8.5-fold higher risk of developing psychosis and schizophrenia, respectively [6]. Although we have a rather limited understanding whether psychosis presented in individuals with schizophrenia is a risk factor of epilepsy, Mäkikyrö et al. reported that epilepsy was strongly associated with schizophrenia (OR = 11.1, 95% CI = 4.0–31.6) in a 28-year follow-up study of the general population in northern Finland; thus, it is plausible that individuals with schizophrenia are susceptible to epilepsy [11]. Moreover, the association with epilepsy is not limited to schizophrenia, but other psychiatric disorders as well such as acute stress disorder, anxiety, depression, bipolar disorder, attention deficit hyperactivity disorder, sleep disorders, and movement disorders [5, 12–16].

Epilepsy can be categorized into generalized or partial epilepsy. Generalized epilepsy is a widespread seizure which affects the entire brain, whereas partial epilepsy is limited to a particular region of the brain. Temporal lobe epilepsy (TLE) belongs to partial epilepsies, mainly originating from a seizure in the hippocampus. In relation to psychiatric illness, recent meta-analysis of systematic review demonstrated that the estimated prevalence of psychosis was higher among individuals with TLE, implicating that the temporal lobe area may be a key brain region, sharing the pathophysiology of TLE and psychosis [17]. In addition to TLE, febrile seizure is worthwhile to discuss as it may have a common pathology, relative to schizophrenia. Febrile seizures, convulsions triggered by a fever, are a fairly common malady occurring in young children between the age of 6 months and 5 years with a 2–14% prevalence [18]. Although most cases of febrile seizures are benign and leave no subsequent brain damage, it is noteworthy that 30–70% of individuals with TLE have experienced febrile seizures during their childhood [19, 20]. More interestingly, it has been reported that children with a history of febrile seizures have a 44% increased risk of schizophrenia [21]. Febrile seizures occur in children when their brains are developing and have high plasticity. Coincidentally, the onsets of schizophrenia as well as TLE frequently occur around adolescence, the final stage of the brain's development. Perhaps, it would be reasonable to speculate that recurrent and prolonged seizures result in alterations to the developing brain architecture, which serves as a trigger to disassemble the molecules involved in forming improper neural networks. Formation of improper neuronal networks could be deleterious to brain functionality, leading to devastating psychiatric conditions. In the following sections, we discuss pathophysiological evidence supporting such ideas, especially focusing on the hippocampal neuronal circuits. Abnormal anatomical and functional structures of hippocampi found in both individuals with TLE and schizophrenia further suggest potential association in these disease states [22–25].

## 2. Hippocampal Dysfunction Is an Overlapping Feature of Temporal Lobe Epilepsy and Psychiatric Disorders

The hippocampus is the largest structure of the mesial temporal lobe and believed to be the primary brain structure underlying the pathophysiology of hallucinations and disturbance of cognition, both of which are symptoms common to TLE and schizophrenia. In translational studies on individuals with TLE, electroencephalogram recordings as well as electrophysiological characterizations showed synchronous hyperactivity and the presence of spontaneously occurring interictal spike discharge in the hippocampus [26–28]. Similarly, in individuals with schizophrenia, brain imaging analyses using positron emission tomography and functional magnetic resonance imaging indicated an abnormally hyperactive hippocampus, which coincided with elevated cerebral blood flow, indicating an increase in basal metabolism [29–37].

It is of note that hippocampal hyper metabolism appears after the onset of psychosis and predicts subsequent atrophy of the hippocampus [38]. Intriguingly enough, this neuroimaging study, together with the ketamine-induced animal model of schizophrenia, implicates that elevation in extracellular glutamate triggers hippocampal hypermetabolism and atrophy, both of which are pathogenic abnormalities related to psychosis [38].

Glutamatergic dysfunction is becoming a well-accepted concept underlying the pathophysiology of schizophrenia, although it is largely elusive exactly how altered glutamate signaling initiates and manifests schizophrenic illness. Consistent with this observation, hippocampal postmortem brain analyses from individuals with schizophrenia revealed elevated expression of brain-derived neurotrophic factor (BDNF) mRNA as well as GluN2B-containing NMDA receptors, and postsynaptic density protein 95 (PSD-95) proteins, particularly in the CA3 subregion, all of which indicate an increase in synaptic strength through, in part, increasing levels of molecules involved in glutamatergic synaptic transmission [39–41]. Similar to the molecular changes observed in schizophrenic individuals, a number of studies using hippocampal autopsies from patients with TLE demonstrated increase in BDNF mRNA and protein levels of AMPA and NMDA receptor subunits, implicating enhanced excitatory synaptic transmission [42–44]. Moreover, in TLE, hyperexcitability in the hippocampus is also seen during the process of epilepsy development and can contribute to an epileptogenic focus by inducing atrophy of the hippocampus (see review [45–49]). Altogether, both TLE and schizophrenia display similar dysfunctionality, particularly aberrant excitatory synaptic transmission, within the hippocampus.

## 3. Ectopic Mossy Fiber Projection Represents Pathophysiology of Temporal Lobe Epilepsy

The hippocampus consists of three major subfields: the dentate gyrus (DG) and the Cornus Ammonis areas 1 and 3 (CA1 and CA3) subfields (Ammon's horns), forming a unidirectional network via a trisynaptic circuit. Within the hippocampal circuit, the DG is an entry point which receives the afferents from, but not limited to, the entorhinal cortex, via the so called perforant pathway. Axons of granule cells in the DG, often referred to as mossy fibers, project to the CA3 through the hilus of the DG and make contact with pyramidal cells in the CA3, forming the DG-CA3 circuit. Pyramidal cells of the CA3 then extend their axons to the CA1. Thus, the perforant pathway, the DG-CA3, and the CA3-CA1 circuits compose the trisynaptic circuit of the hippocampus. Within the trisynaptic circuit, the DG-CA3 connection, also known as the mossy fiber pathway, could serve as a regulator of the net hippocampal activity since the DG is the primary subfield in the hippocampus, receiving exogenous inputs from other brain regions.

**Figure 1:**
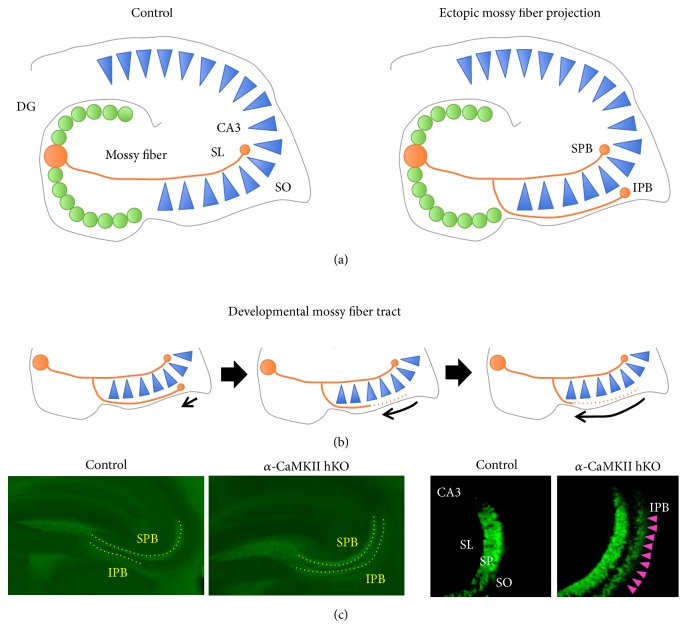
(a) Under normal conditions, mossy fibers and axons of granule cells target the SL region in the CA3 subfield, forming SPB. Contrastingly, a pathological condition occasionally results in ectopic mossy fiber projection, in which axons are guided not only to the SL, but also to the SO. (b) During postnatal development, both SPB and IPB are formed. Mossy fibers projected to the SO undergo retraction, thus leaving only SPB as they mature. (c) Presented are immune fluorescent staining of Znt-3 in the hippocampal sections. In *α*-CaMKII hKO mice, mossy fibers display robust projection onto the SO, developing IPB as indicated by arrowheads.

In adults, mossy fibers normally project to proximal apical dendrites of pyramidal cells in the stratum lucidum (SL) of the CA3 (Figure 1(a)), whereas, during postnatal development, mossy fibers from the immature granule cells extend to both the SL and stratum oriens (SO), forming both suprapyramidal bundles (SPB) and infrapyramidal bundles (IPB), respectively (Figure 1(b)). The formation of IPB, however, is transient as stereotyped pruning occurs via axon retraction that is triggered by Plexin A3 signaling [50, 51]. The pruning of inappropriate synaptic contacts formed by mossy fiber is a critical step to mature the DG-CA3 network during development. It is of interest that in response to extrinsic stimuli, newly born neurons in adults will shape preferentially, but not exclusively, IPB, further emphasizing the importance of pruning [52].

Maintenance of the proper mossy fiber pathway is necessary for normal hippocampal function throughout life as granule cells from the DG are continuously born and incorporated into the hippocampal network. In an animal model of epilepsy, abnormal axon collaterals and branches produced from a main axon have been observed within the hilus of the DG [53–56]. The abnormal collaterals ectopically innervate from the hilus to the molecular layer of the DG, making contacts with apical dendrites of granule cells and forming extra excitatory synapses. This abnormal synaptic morphology is called mossy fiber sprouting, which occurs not only in animal models of epilepsy, but also in individuals with TLE and bipolar disorder [57–61, 76]. In epileptic hippocampi, abnormal mossy fiber projection has also been reported in the SO of the CA3 subregion, somewhat similar to the developmental state of mossy fiber maturation. It is reported that seizures induced by kainate acid treatments enhanced the number of newly born granule cells in the DG, which subsequently resulted in increased formation of IPB in the SO along with hyperactivity [52]. Although the role of aberrant mossy fiber projection is unclear, one suggests that synapses produced by mossy fiber sprouting are functionally active, because axon selection depends on excitatory pre- and postsynaptic activity, resulting in hyperexcitation of the DG and the CA3, thus, the origin of epileptogenesis [45–49].

## 4. Rodent Models of Schizophrenia Display Ectopic Mossy Fiber Guidance

Similar to an epileptic CA3, we recently identified that mossy fibers were ectopically guided to the SO of the CA3 subfield, forming IPB in mice with heterozygous knockout of *α*-isoform of calcium/calmodulin-dependent protein kinase II (*α*-CaMKII hKO), one of the rodent models of schizophrenia. *α*-CaMKII hKO is a well-studied kinase for its role in learning, memory, and its electrophysiological correlate, long term potentiation. This *α*-CaMKII hKO mouse displayed impaired working memory, social interaction, and locomotion activity that are reminiscent of clinical symptoms presented by schizophrenia [62] (Figures 1 and 3). The abnormal morphology of mossy fiber identified in *α*-CaMKII hKO mice is not only a morphological phenotype but also linked to alterations in electrophysiological properties of field excitatory postsynaptic potentials at mossy fiber-CA3 synapses [63]. Hippocampal slices from *α*-CaMKII hKO mice revealed increased basal transmission from the mossy fiber terminals as measured by field recording and increased neuronal activity in MRI study in CA3 and in CA1 [63, 64] that matches to the observed hyperactivity in the hippocampus via CBV study in schizophrenia [33, 38, 65, 66]. Given the human fMRI study that the hyperactivity in the CA3 resulted in the hyperactivity in the CA1 [67], it is plausible that the mossy fiber misguidance triggers the hyperactivity in the CA3 and then results in the hyperactivity in the CA1 as well as whole hippocampus. In addition to *α*-CaMKII hKO mice, the abnormal morphology of mossy fiber has been reported in other genetic models of schizophrenia in rodents. Disrupted-in-Schizophrenia-1 (DISC1) is a susceptibility gene for schizophrenia based on genetic linkage and association studies [68]. Adult mice with retro-virus-mediated knockdown of DISC1 displayed ectopic axonal guidance of newborn mossy fibers, which extended beyond the SL of the CA3 subfield and overshot to the CA1 subfield [69]. Furthermore, DISC1 knockdown led to accelerated maturation of mossy fiber boutons. Defects in mossy fiber also have been found in mice lacking the synaptosomal-associated protein of 25 kDa (SNAP25) gene, which is significantly associated with several psychiatric disorders including schizophrenia [70]. In neoexcised SNAP-25b deficient mice, mossy fibers are enlarged in the CA3 area [71]. Taken together, it is reasonable to say that abnormal mossy fiber formation is commonly found in rodent models of schizophrenia. To date, however, little is known about how these gene deletions lead to abnormality in mossy fiber projections. In the following section, we speculate the possible mechanism based on the knowledge of cellular and molecular characters that we identified in *α*-CaMKII hKO mice.

## 5. Possible Molecular and Cellular Mechanism Underlying Ectopic Mossy Fiber Guidance

**Figure 2:**
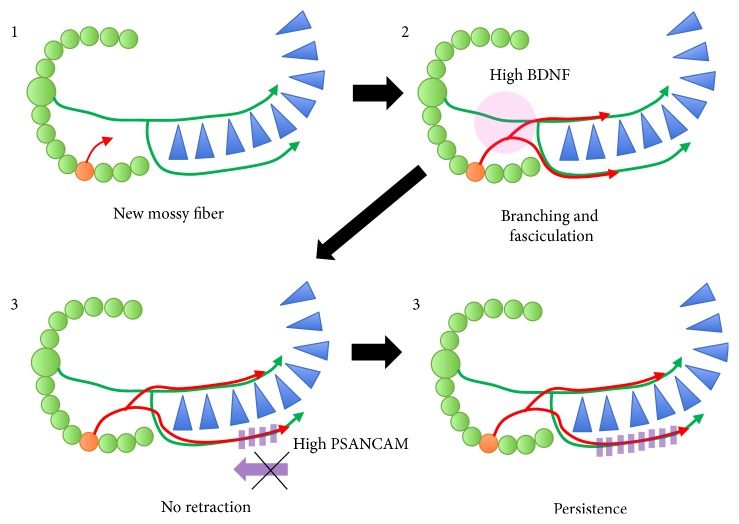
Upon maturation of newborn granule cells in neurons, excess amounts of BDNF possibly induce branching of mossy fibers towards the SO, and exacerbated fasciculation may occur in *α*-CaMKII hKO mice. Retraction of mossy fiber axons in the SO fails due to high expression of PSA on NCAM, thus displaying persistent presence of ectopic mossy fiber projection.

The hippocampus in *α*-CaMKII hKO mice showed 30% upregulation in BDNF compared to wild-type mice [63]. BDNF has been shown to be necessary and sufficient to promote hyperactivity-induced mossy fiber sprouting in hippocampal slice cultures [77]. Mice overexpressing BDNF in the forebrain displayed structural alterations in the mossy fiber pathway, including an enlarged infrapyramidal compartment [78]. Based on these evidences, the ectopic mossy fiber guidance observed in *α*-CaMKII hKO mice could be mediated through elevated BDNF levels in the DG (Figure 2). Importantly, BDNF expression is regulated in an activity-dependent manner [79], suggesting that mossy fiber sprouting could occur upon neuronal activation. Under disease conditions in schizophrenia and TLE, hippocampal hyperactivity is frequently observed, which could induce mossy fiber sprouting. Consequently, aberrant mossy fiber sprouting could lead to the increased formation of synapses in the CA3 subregion, further enhancing synaptic transmission. Thus, these seemingly unrelated features of neurons, mossy fiber sprouting, and hippocampal hyperactivity regulate reciprocally and constitute feed-forward regulation within hippocampal circuits. Perhaps such type of regulation may contribute to the progressive nature of symptoms in schizophrenia and TLE. In addition to BDNF, polysialic acid neural cell adhesion molecule (PSA-NCAM) is another candidate to guide mossy fiber and is highly present in the DG of *α*-CaMKII hKO mice [63]. NCAM is a transmembrane protein essential for cell-to-cell interaction and is involved in cell migration and axon guidance during development as well as synaptic transmission and cognitive function in adult brains [45, 80]. Importantly, NCAM undergoes polysialylation, an attachment of PSA moiety, which intricately mediates the axon guidance of newly born neurons and establishes functional synaptic connections at a discrete region in the hippocampus. The presence of PSA moiety on NCAM appeared to be necessary for the appropriate innervation of mossy fibers, as enzymatic and genetic removal of PSA resulted in excessive defasciculation of mossy fibers [81, 82]. Thereby, PSA expression on NCAM is thought to be a contact-mediated mossy fiber guidance cue. The fact that PSA-NCAM expression is robustly elevated in *α*-CaMKII hKO mice could imply the reinforced fasciculation of mossy fibers, inhibiting retraction of misguided axons at the SO (Figure 2). As a chemorepulsive factor, Sema3A also contributes to ectopic mossy fiber projection. Sema3A, a secreted factor known to repel axon guidance, is increased in the developmental stage and prevents mossy fiber outgrowth in the SO region [83], while knockdown of Sema3A signaling maintains the mossy fiber subfield in the SO [50, 51]. In *α*-CaMKII hKO mice, the Sema3A expression is decreased in the hippocampus, suggesting the involvement of reduced Sema3A in the mispathfindings of the mossy fiber [62].

A key cellular feature of *α*-CaMKII hKO mice in relation to the pathophysiology of schizophrenia is “immature dentate gyrus (iDG),” which is characterized by an increase in calretinin-positive immature neuronal progenitors and a decrease in calbindin-positive mature neurons in the hippocampus. Importantly, the iDG-like phenotype was immunohistochemically detected in postmortem brain samples from individuals with schizophrenia [74]. In support of the immunohistochemical finding, microarray analysis of the DG from a postmortem schizophrenic human brain detected a significantly decreased expression of calbindin, a maker of mature neurons [84]. Moreover, when the expression of calretinin and the immature maker was examined with the clinical data for schizophrenia, positive correlation was found with suicide death, psychosis, and duration of disease. Taken together, the above findings underscore iDG as a hallmark which links a rodent model to the human conditions of schizophrenia. Although we have limited understanding how newly born neurons are improperly incorporated into hippocampal circuits with iDG in *α*-CaMKII hKO mice, it is plausible that ectopic mossy fibers originate from the erratic axon guidance of accumulated immature neurons. To support this idea, ectopic mossy fiber projection at the SO can be transiently seen only in immature neurons in normal rodents and gradually undergoes its pruning during development [50, 51], suggesting that immature neurons have ectopic mossy fiber projection.

Although one paper showed the density of mossy fiber was reduced in the stratum lucidum in the CA3 region in patients with schizophrenia [85], so far there is no report which examined mossy fiber pathfindings (the projection site) in the stratum radiatum and oriens region in the schizophrenia. However, given these evidences we have the following. (1) Immaturation of granule cells in the DG was observed both in *α*-CaMKII hKO animals and in patients with schizophrenia [63, 74]. (2) It is reported that the mossy fiber misguidance is the reflection of immaturation of granule cells [50, 51]. (3) *α*-CaMKII hKO animals have the mossy fiber misguidance. (4) The synaptic density in the stratum radiatum was increased in patients with schizophrenia [39]. (5) The number of postsynaptic synapse is correlated with the number of the mossy fiber synapse [86]; it suggests that the possibility that mossy fiber increased their subfield outside stratum lucidum. Further effort to identify mossy fiber misguidance with immunohistochemistry in postmortem brain in patients with schizophrenia is currently underway in our laboratory.

As we discussed earlier, structural alterations within the mossy fiber pathway could be a pathophysiological feature shared by epilepsy and schizophrenia. Further extending this notion, we recently demonstrated common cellular, molecular, and behavioral phenotypes in rodent models of epilepsy and schizophrenia [87]. To induce a seizure, animals were challenged with a single dose of pilocarpine and examined behaviorally and cellularly. This paradigm is well known to represent the pathophysiology of TLE [88]. The animals treated with pilocarpine exhibited an increased expression of calretinin and decreased expression of calbindin in the DG, both of which are principal features of the iDG-like phenotype identified in *α*-CaMKII hKO mice. Importantly, reduced expression of calbindin within the hippocampal granule cells has reportedly been found in the postmortem brain of individuals suffering from TLE [75]. BDNF and PSA-NCAM expressions are also upregulated, while Sema3A and *α*-CaMKII hKO show a decrease in the hippocampus in models of individuals with TLE [89–95]. The iDG-like phenotype in the animals treated with pilocarpine coincided with increased locomotor activity, poor working memory formation, and impaired social interaction, all of which are core behavioral deficits observed in *α*-CaMKII hKO mice [87]. Also, it is of note that pilocarpine treatment in *α*-CaMKII hKO mice decreased the threshold of seizure induction. In an electrophysiological study, *α*-CaMKII hKO mice and pilocarpine-treated mice had similar characteristics in the DG, such as lowered resting potential, all of which suggest the shared characteristics of abnormal mossy fiber projection along with iDG phenotype and its outcome both in the epileptic and in the schizophrenic brain.

## 6. ***α***-CaMKII as a Potential Therapeutic Target in Future Drug Discovery

**Figure 3:**
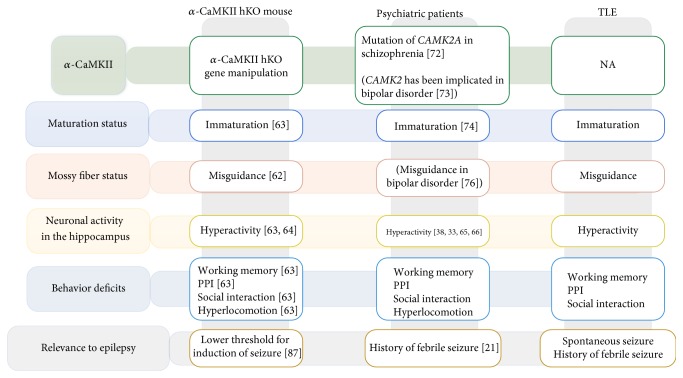
A schematic representation of the shared pathophysiology from genetic level to behavior level across *α*-CaMKII hKO mice, psychiatric patients, and patients with TLE. A mutation of CAMK2A gene was found in patients with schizophrenia and it is also implicated in bipolar disorder [72, 73]. *α*-CaMKII hKO mice have behavioral deficits [63] which are observed in patients with schizophrenia and TLE [74, 75]. Furthermore, *α*-CaMKII hKO animals were shown to have immaturation of granular cells in DG which was also observed in patients [74]. Importantly, the *α*-CaMKII hKO mouse showed the neuronal hyperactivity [63, 64] that matches to the observed hyperactivity in the hippocampus in patients [33, 38, 65, 66]. These shared features and the having history of febrile seizure in patients lead us to hypothesize the presence of mossy fiber misguidance [76] in patients with schizophrenia.

In the present review, we have pointed commonality and a pivotal role of *α*-CaMKII in pathophysiology of TLE and schizophrenia (Figure 3). Intriguingly, a recent genetic study identified nonsense mutations within CAMK2A gene in patients with schizophrenia [72], further supporting possibility of CaMKII as an indispensable molecule to mediate pathophysiological conditions of schizophrenia. Given its protein function, *α*-CaMKII itself could be a direct therapeutic target; however, *α*-CaMKII is ubiquitously expressed throughout the body, which may hinder interventions to regulate *α*-CaMKII's activity specifically in the brain. Rather the use of *α*-CaMKII hKO mice would be advantageous to investigate molecular mechanisms underlying pathophysiological states reminiscent to TLE and schizophrenia. Profiling gene expression followed by pathway analysis would be one approach to pinpoint a disturbed pathway, allowing us to select druggable targets. Alternatively, identification of substrates for *α*-CaMKII may direct us to novel intervention. Much more investigations warrants the *α*-CaMKII hypothesis and its targets in drug discovery.

## 7. Conclusion

It is becoming evident that TLE and schizophrenia share commonality in various aspects, leading us to postulate similar etiology of these disorders. In fact, clinical trials are ongoing to testify to the effect of antiepileptic drugs for patients with schizophrenia (https://clinicaltrials.gov/ct2/show/NCT03034356) via reducing hippocampal abnormal neuronal activity. To further support this idea, we have extensively investigated *α*-CaMKII hKO mice, which not only display behavioral phenotypes reminiscent of clinical presentations of schizophrenic individuals but also are prone to epilepsy. Ectopic mossy fiber path finding, a novel finding identified in *α*-CaMKII hKO mice, is a cellular phenotype that is also reported in epileptic brains. Relative to glutamate dysfunction believed to underlie negative symptoms and cognitive deficits of schizophrenia, ectopic mossy fiber guidance is likely another contributing factor, resulting in dysregulated excitatory synaptic transmission within the hippocampal circuits, particularly in the CA3 subfield. Identification of molecular components, as well as its mechanism of mossy fiber guidance, potentially provides a new avenue for therapeutic interventions of schizophrenia that is an alternative to classical antipsychotic drugs. Furthermore, outside of schizophrenia, mechanistic understanding of ectopic mossy fiber pathfinding will benefit the intervention of epileptic conditions.
